# Wireless Transmission Method for Large Data Based on Hierarchical Compressed Sensing and Sparse Decomposition

**DOI:** 10.3390/s20247146

**Published:** 2020-12-13

**Authors:** Youtian Qie, Chuangbo Hao, Ping Song

**Affiliations:** 1 The Key Laboratory of Biomimetic Robots and Systems, Ministry of Education, Beijing Institute of Technology, Beijing 100081, China; youtianqie@bit.edu.cn (Y.Q.); haochuangbo@sina.com (C.H.); 2 The Beijing Jinghang Computation and Communication Research Institute, Beijing 100074, China

**Keywords:** wireless transmission method, wireless sensor network, compressed sensing, Orthogonal Matching Pursuit, signal sparsity

## Abstract

With the widespread application of wireless sensor networks, large-scale systems with high sampling rates are becoming more and more common. The amount of original data generated by the wireless sensor network is very large, and transmitting all the original data back to the host wastes network bandwidth and energy. This paper proposes a wireless transmission method for large data based on hierarchical compressed sensing and sparse decomposition. This method includes a hierarchical signal decomposition method based on the same sparse basis and different sparse basis hierarchical compressed sensing method with a mask. Compared with the traditional compressed sensing method, this method reduces the error of signal reconstruction, reduces the amount of calculation during signal reconstruction, and reduces the occupation of hardware resources. We designed comparison experiments between the traditional compressed sensing algorithm and the method proposed in this article. In addition, the experiments’ results prove that our proposed method reduces the execution time, as well as the reconstruction error, compared with the traditional compressed sensing algorithm, and it can achieve better reconstruction at a relatively low compression ratio.

## 1. Introduction

With the widespread application of wireless sensor networks and the Internet of Things, the collecting requirements of physical parameters are increasing exponentially. The scale of wireless sensors networks is increasing, and the amount of data carried by wireless sensor networks is increasing. The bandwidth of wireless networks is increasingly unable to carry such a huge amount of data. Therefore, we urgently need to determine a compression method suitable for wireless sensor networks to manage the huge amount of data.

**Figure 1:**
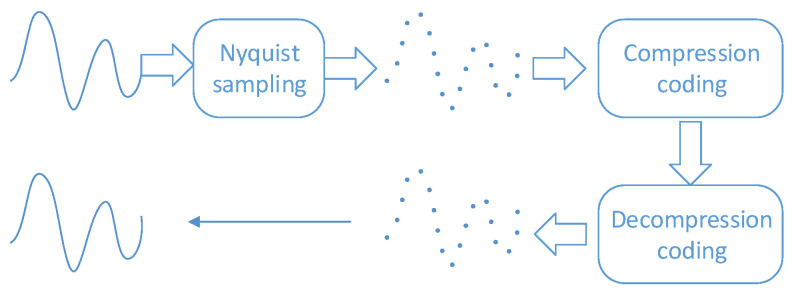
The traditional compression and decompression process.

According to the Nyquist sampling law, when the minimum sampling frequency is at least twice the highest frequency of the signal, frequency aliasing will not occur when the signal is restored. However, a high sampling rate will produce more redundant data for signals with strong correlation. In wireless sensor networks, data compression coding is required to remove redundant data after sampling. The traditional compression and decompression process is shown in Figure 1.

**Figure 2:**
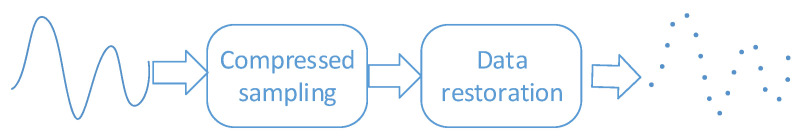
The process of compressed sensing.

The compression algorithm of commercial compression software is generally lossless compression, but their compression effect on data is relatively poor. Sparse transform also has the function of data compression, and the compression effect is not bad. However, because the wireless sensor network is unstable, packet loss often occurs. Sparse transformation is more sensitive to lost data, and lost data has a greater impact on signal reconstruction, so it is not suitable for wireless sensor networks. Compressed sensing algorithms, as an effective new data compression method, were proposed by Candes et al. [1,2] and Donoho [3], who provided strict mathematical proofs. Provided the signal is sparse in a certain orthogonal space, the signal can be sampled at a low frequency, and the signal can be reconstructed with high probability. This theory breaks through the limitations of the Nyquist sampling theorem and has been successfully applied in many fields, such as image, radar, wireless sensor network, and remote sensing [4,5,6,7,8,9]. The process of compressed sensing as shown in Figure 2.

Traditional compressed sensing is insensitive to data packet loss in wireless networks, and has the advantages of automatic encryption, high compression rate, small reconstruction error, but it also has disadvantages such as large resource usage and large calculation amount.

Based on the above research, this paper hence proposes a Wireless transmission method for large data based on hierarchical compressed sensing and sparse decomposition. The main contributions of this paper are summarized as follows.

1. In this paper, we propose a wireless transmission method for large data based on hierarchical compressed sensing and sparse decomposition. Compared with the traditional compressed sensing method, this method reduces the error of signal reconstruction, reduces the amount of calculation during signal reconstruction, and reduces the occupation of hardware resources.
2. In this paper, we propose a hierarchical decomposition method based on the same sparse basis and different sparse bases. This method can select appropriate sparse bases for sparse decomposition according to the characteristics of the signal, and can reduce the reconstruction error of the signal under the same compression ratio.
3. In this paper, we propose a hierarchical compressed sensing method with a mask (HCSWM). This method greatly reduces the amount of calculation and resource occupation through hierarchical compression, hierarchical reconstruction and a mask. In addition, this paper analyzes the effect of this method on reducing the amount of calculation from a theoretical point of view, and finally designed a comparative experiment to prove the effect.

The remainder of this paper is organized as follows. Section 2 introduces the related work of existing compressed sensing algorithms, Section 3 proposes the wireless transmission method for large data based on hierarchical compressed sensing and sparse decomposition, Section 4 presents comparative experiments between the traditional compressed sensing algorithm and the method proposed in this article, Section 5 analyzes the results of the experiment, and give a deep analysis of the reasons, Section 6 summarizes the conclusion of the paper.

## 2. Related Work

### 2.1. Traditional Compressed Sensing Algorithm

The essence of compressed sensing is to use the sparsity of the signal in a certain transform domain [10]. As long as the original signal satisfies the “sparse” condition, an observation matrix that is not related to the sparse transformation basis can be used to project the original high-dimensional sequence into a low-dimensional space to achieve the purpose of reducing the dimensionality of the source signal. Assuming that the original signal $O={\left[o_{1},o_{2},,,o_{N}\right]}^{T},O\in R^{N},$ is compressible. Then *O* needs to be sparse by itself or sparse after sparse basis conversion. The sparse basis conversion is shown as

(1) $$\begin{array}{c} O=\sum^{N}\psi_{i}{\hat{o}}_{i}=\Psi \hat{O} \end{array}$$

where Ψ is the sparse basis and $\hat{O}$ is sparse representation of the signal. Next, the sparse signal $\hat{O}$ needs to be projected to an observation matrix $\Phi \in R^{M\times N}$ that is not related to the sparse basis. To ensure that the original signal can be reconstructed through signal restoration, the observation matrix Φ needs to satisfy the Restricted Ismoetry Property (RIP) [1], as shown

(2) $$\begin{array}{c} \left(1-\delta_{K}\right)\left|\right|\hat{O}{\left|\right|}_{l2}^{2}\leq ||\Phi \Psi \hat{O}{\left|\right|}_{l2}^{2}\leq \left(1-\delta_{K}\right)\left|\right|\hat{O}{\left|\right|}_{l2}^{2} \end{array}$$

If the equidistant constant $\delta_{K}$ is less than and not close to 1, the observation matrix Φ satisfies the RIP property [11]. The irrelevance to the sparse basis Ψ is also to ensure RIP conditions [3]. Compressed signal *C* after dimensionality reduction is as shown

(3) $$\begin{array}{c} C=\Phi O=\Phi \Psi \hat{O} \end{array}$$

Finally, restored signal *Y* is obtained through the optimization problem under the 0-norm to achieve the purpose of restoring the original signal, as shown

(4) $$\begin{array}{c} min||\Psi^{T}Y{\left|\right|}_{0}\;s.t.\Phi Y=C \end{array}$$

Since M<N, the objective optimization function is an ill-conditioned equation, and the solution of the equation is not unique. To optimize the solvability of the equation, the 0-norm in the optimization function is often converted to 1 norm [12,13]. In reality, when Φ and Ψ can satisfy the irrelevant properties, there is a high probability that the above-mentioned signals can be recovered accurately, as shown

(5) $$\begin{array}{c} min||\Psi^{T}Y{\left|\right|}_{1}\;s.t.\Phi Y=C \end{array}$$

### 2.2. Sparse Representation of the Signal

The requirement of compressed sensing is that the signal has sparsity [14]. The sparsity of the compressed sensing signal includes two cases: the original signal *O* itself is a sparse signal, as in Equation (6), or the original signal is a sparse signal in a sparse basis projection, as shown in Equation (7), the signal *O* has $K_{\mathrm{sp}}$ sparsity.

(6) $$\begin{array}{c} {\left||O|\right|}_{0}=K_{\mathrm{sp}}\;\left(K_{\mathrm{sp}}<<N\right) \end{array}$$

(7) $$\begin{array}{cc} O= & \Psi \hat{O}\\ \left|\right|\hat{O}{\left|\right|}_{0}= & K_{\mathrm{sp}}\;\left(K_{\mathrm{sp}}<<N\right) \end{array}$$

In practice, wireless sensor networks in particular deal with physical quantities in nature, and most of these signals are not strictly sparse but approximately sparse. If the elements of the vector signal *O* are sorted by amplitude and the amplitude attenuation speed has a power speed, then the signal *O* is called Ψ domain compressible [1]. In the implementation of compressed sensing algorithms, approximate sparse transformation is generally performed. The method of threshold or mask is used for sparse approximate representation. At this time, compressed sensing is a lossy compression method, but it also has a certain filtering effect on the signal.

Determining the sparse basis of the signal is an important research content in the field of compressed sensing. Most of the physical quantity signals monitored by wireless sensor networks can be sparsely transformed with Fourier transform basis, cosine transform basis, wavelet transform basis, and Gabor transform basis; For image signals, cosine transform basis and Curvelet transform basis can be used for sparse transformation. For other more complex signals, a redundant dictionary can be constructed to replace orthogonal basis, and the linear combination of atoms in the dictionary can be used to represent the signal, and the signal can be sparsely approximated or nonlinearly approximated [15,16,17].

### 2.3. Uncorrelated Observation Matrix Design

To ensure that the signal can be restored without distortion within a certain error range, the observation matrix needs to meet the RIP property, When the observation matrix Φ is not related to the transformation basis Φ, the observation matrix satisfies the RIP property with a high probability [18]. When designing, ensure that the observation matrix and the sparse signal are as uncorrelated as possible. The commonly used measurement matrices are: Gaussian random matrix (Equation (8)) and Bernoulli matrix (Equation (9)). Gaussian random matrix is the most common measurement matrix in compressed sensing. However, the calculation is complicated and not suitable for wireless sensor networks. The projection calculation of Bernoulli matrix is relatively simple, and it is widely used in hardware implementation. Therefore, Bernoulli matrix is used as the observation matrix in this article.

(8) $$\begin{array}{c} \Phi_{i,j}=N\left(0,1/\sqrt{M}\right)1\leq i\leq M,1\leq j\leq N \end{array}$$

(9) $$\begin{array}{c} \Phi_{i,j}=\left\{\begin{array}{cc} 0 & p\;=\;0.5\\ 1 & p\;=\;0.5 \end{array}\right. \end{array}$$

### 2.4. Common Signal Restoration Algorithms

The essence of the signal reduction problem is to solve an ill-conditioned equation of Equation (5), which is equivalently transforming the original objective function formula into a convex optimization problem. At present, there are many kinds of related reconstruction algorithms, which can be roughly divided into three categories:

- Greedy pursuit algorithm: The basis of this type of algorithm is Orthogonal Matching Pursuit (OMP) [19], This method calculates the correlation coefficient of the residuals, selects the atoms in the observation matrix, and updates the residuals after projection of the residuals to perform iteration. This method is suitable for applications with high real-time requirements. This article uses OMP algorithm as a reconstruction method.
- Convex optimization algorithm: This algorithm regards the problem as a convex optimization problem and successively approximates the original signal. Its advantage is high reconstruction accuracy, but its disadvantage is high algorithm time complexity [20].
- Combination algorithm: This type of algorithm combines the above two methods, requiring that the signal can be reconstructed in groups, and the performance is somewhere between the two [21].

## 3. Wireless Transmission Method for Large Data Based on Hierarchical Compressed Sensing and Sparse Decomposition

### 3.1. Architecture Design

In theory, compressed sensing can achieve high-frequency signal testing through a lower sampling rate. However, because the projection of the original signal needs to be calculated in Equation (5), it is difficult to perform the projection operation when the original signal is unknown. Baraniuk et al. [22] of RICE University proposed an analog-to-signal converter (AIC) compression sensing circuit for high-frequency signals . The signal generated by the DAC is mixed with the original signal to realize signal projection on the analog circuit. However, in wireless sensor networks, node hardware resources and energy are limited, and it is difficult to implement hardware circuits such as DAC circuits and mixers in AIC circuits. If the hardware is not changed, the method of encoding and compressing after acquisition is generally used. It just uses compressed sensing to do the projection of the observation matrix during compression coding. Although this will not reduce the amount of data collection, and increase the amount of processing and calculation of the node, it can greatly reduce the amount of data transmission in the process of wireless sensor network data transmission, and increase the network’s robustness after eliminating redundant data. The robustness makes the network insensitive to packet loss and effectively reduces the amount of data transmission. Since the energy consumption of data transmission accounts for about 90% of the total energy consumption, reducing the amount of data transmission at the cost of increasing the calculation of nodes can save network bandwidth, reduce energy consumption, and increase node life. Currently, most wireless sensor networks use this method for compressed sensing data transmission [23,24]. When compressed sensing is used to eliminate data redundancy for signals, it is also a filtering process to filter out the secondary signals in the primary sparse basis. Therefore, compressed sensing is mostly a lossy compression process in practical applications.

**Figure 3:**
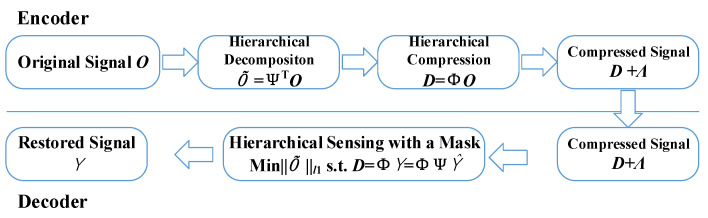
Architecture design of Wireless transmission method for large data.

Wireless transmission method for large data based on hierarchical compressed sensing and sparse decomposition mainly include a hierarchical signal decomposition method based on the same sparse basis and different sparse basis, hierarchical compressed sensing method with a mask (HCSWM). The HCSWM mainly includes hierarchical compression and the orthogonal matching pursuit reconstruction algorithm based on a mask. The wireless transmission method of the wireless network test node is divided into an encoding end and a decoding end, as shown in Figure 3. The encoder mainly includes a hierarchical signal decomposition method based on the same sparse basis and different sparse basis and hierarchical compression, and decoder mainly includes the orthogonal matching pursuit reconstruction algorithm based on a mask. The encoder is located on one or more nodes in the wireless network test, and the decoding end is located on the upper computer terminal. The function of the encoder is to convert the original *N*-dimensional signal *O* into an *N*-dimensional sparse signal $\hat{O}$ through the sparse transformation of Ψ, and obtain the *M*-dimensional measurement value through the projection of the M×N-dimensional observation matrix Φ. The function of the decoder is to accurately reconstruct the original signal by solving the problem of minimum L1 norm optimization after receiving the measured value.

### 3.2. Hierarchical Decomposition Method Based on the Same Sparse Basis and Different Sparse Bases

#### 3.2.1. Prepare the Signal Source

**Figure 4:**
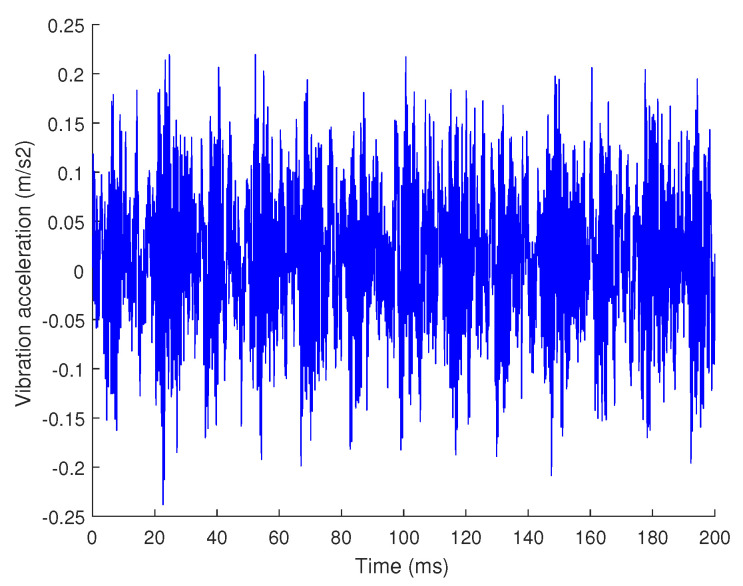
Vibration signal.

**Figure 5:**
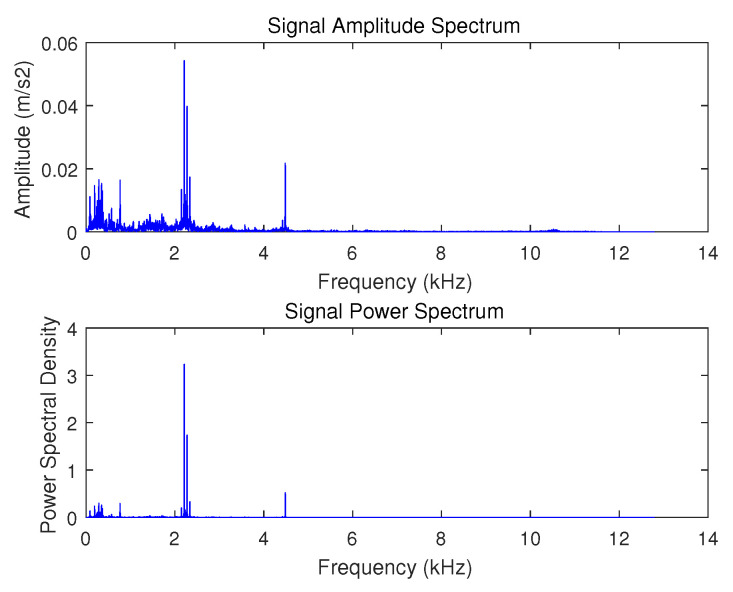
Amplitude spectrum and power spectrum.

In the real environment, most of the physical quantities collected in nature are not strictly sparse. For example, Figure 4 shows a time domain signal of vibration acceleration(The data are from the bearing dataset of the Western Reserve University) The amplitude spectrum and power spectrum of the signal after sparse transformation are shown in Figure 5. It can be seen that in the amplitude spectrum and power spectrum, most of the frequency signals are 0 or close to 0, and only a few frequencies have larger values.

#### 3.2.2. Hierarchical Decomposition Method with the Same Sparse Bases (HDWSS)

The observation matrix in Equation (3) needs to satisfy Equation (10) to better restore the original signal [3,20]. Since the number of rows of the observation matrix Φ is the length of the signal after dimensionality reduction, we can use Equation (11) to calculate the maximum compression ratio of compressed sensing.

(10) $$\begin{array}{c} M\geq cK_{\mathrm{sp}}\log\left(N/K_{\mathrm{sp}}\right) \end{array}$$

(11) $$\begin{array}{c} R_{c}=M/N\geq cK_{\mathrm{sp}}\log\left(N/K_{\mathrm{sp}}\right)/N \end{array}$$

where *N* is the length of the original signal, $K_{\mathrm{sp}}$ is the sparsity of the signal, and *M* is the rank of the observation matrix after dimensionality reduction. Since *c* is a constant and $1\leq K_{\mathrm{sp}}<<N$, Equation (11) can be written as

(12) $$\begin{array}{cc} cK_{\mathrm{sp}}\log\left(N/K_{\mathrm{sp}}\right)/N= & [cK_{\mathrm{sp}}\log N-cK_{\mathrm{sp}}\log K_{\mathrm{sp}}]/N\\ = & \left(c\log N-c\log K_{\mathrm{sp}}\right)K_{\mathrm{sp}}/N\\ \approx & c\log N\cdot K_{\mathrm{sp}}/N\\ R_{c}= & M/N\geq (cK_{\mathrm{sp}}\log N)/N \end{array}$$

It can be seen that the compression ratio is approximately proportional to the sparsity and is related to the sparsity of the signal. Therefore, when the length of the original signal *N* is constant, we can decompose the signal based on the sparsity, and then decompose the sparse signal into the form of the addition of multiple orthogonal sparse signals, and then compress and encode these signals separately. This method does not affect the compression ratio and compression coding length of the total signal, as shown in Equation (13), and this method can greatly reduce the time of calculation.

(13) $$\begin{array}{cc} K_{\mathrm{sp}}= & K_{\mathrm{sp}1}+K_{\mathrm{sp}2}+\cdots +K_{\mathrm{spn}}\\ R{}_{c}= & (M_{1}+M_{2}+\cdots +M_{n})/N\\ \geq & c\left(K_{\mathrm{sp}1}+K_{\mathrm{sp}2}+\cdots +K_{\mathrm{spn}}\right)\log\left(N\right)/N\\ = & (cK_{\mathrm{sp}}\log N)/N \end{array}$$

where $K_{\mathrm{spi}}$ is the sparsity of the sub-signal, and $M_{i}$ is the rank of the corresponding observation matrix.

Based on the above analysis, we propose the hierarchical decomposition method: first decompose the most important and sparse signal from the total signal, then decompose the less important signal from the residual, and so on. Finally get the decomposed signal *D*. Generally, physical quantities in nature are approximately sparse. Therefore, the signals decomposed at the beginning are often sparse. As the decomposition proceeds step by step, the sparseness of the signal will become worse and the energy will become smaller and smaller. At the same time, the hierarchical signal obtained by this method is more suitable for the transmission of wireless sensor networks with the packet structure. The residual signal *r* can be ignored. The process is as shown

(14) $$\begin{array}{cc} O= & \Psi \hat{D}+r=\Psi \sum_{i}{\hat{D}}_{i}+\Psi \hat{r}\\ D_{i}= & \Psi {\hat{D}}_{i} \end{array}$$

where *r* is the remaining final residual signal, and $\hat{r}$ is the signal after sparse conversion. In order for the information between the sub-signals to be relatively independent, the sparse basis atomic coefficients of the sub-signals are independent of each other, and the correlation between any two sub-signals is 0. Therefore, the sum of the sparsity of each sub-signal is the sparsity of the original sparse signal.

(15) $$\begin{array}{c} {\left||O|\right|}_{l0}=\sum_{i}\left|\right|{\hat{D}}_{i}{\left|\right|}_{l0} \end{array}$$

To achieve the effect of gradual reduction of energy, it is necessary to ensure that the maximum coefficient in each sub-signal is smaller than the minimum coefficient in the previous level during decomposition.

(16) $$\begin{array}{c} \max\left({\hat{D}}_{i}\right)<\min\left({\hat{D}}_{i-1}\right) \end{array}$$

We use the adaptive threshold method to define the value range of ${\hat{O}}_{i}$ for each level.

(17) $$\begin{array}{c} \delta \max\left(\hat{r}\right)\leq {\hat{D}}_{i}\leq \max\left(\hat{r}\right) \end{array}$$

where δ represents the ratio of the lower limit of the signal to the maximum residual signal. The residual signal of the *i*-th level after sparse transformation is shown as

(18) $$\begin{array}{c} {\hat{r}}_{i}=\hat{r}-\sum_{j=1}^{i-1}{\hat{D}}_{j} \end{array}$$

The decomposition steps of the signal are shown in Algorithm 1.

**Algorithm 1** The decomposition steps
**input**: original signal *O*; Layer coefficient δ; Maximum decomposition layer MaxLevel; Minimum stop threshold Ths
**output**: decomposed signal $\hat{D}$
1:
$\hat{O}=\Psi^{T}O$
2:
$\hat{r}=\hat{O},i=0$
3:do4:   for $\hat{r}\left(j\right)\in \hat{r}$5:      if  $\hat{r}\left(j\right)>\delta \max\left(\hat{r}\right)$6:         ${\hat{D}}_{i}\left(j\right)=\hat{r}\left(j\right),\hat{r^{\prime }}\left(j\right)=0,K_{i}++;$7:      else    ${\hat{D}}_{i}\left(j\right)=0,\hat{r^{\prime }}\left(j\right)=\hat{r}\left(j\right);$8:      $\hat{r}=\hat{r^{\prime }},i=i+1$9:while($i<\mathrm{MaxLevel}\;|\;\hat{r}<\mathrm{Ths}$)10:return $\hat{D}$


Traditional signal decomposition, such as frequency domain decomposition [25], wavelet decomposition [26], EMD decomposition [27], etc., is decomposed according to signal attributes such as frequency, scale, and mode, so that the main information contained in the signal may be decomposed into different in scale, frequency or mode. It is not conducive to restore the main body of the signal. For example, a low-frequency and high-frequency mixed signal, the main signal in the frequency domain is decomposed into two parts of low frequency and high frequency; in the discrete wavelet decomposition, it is decomposed into two parts of low-level wavelet and high-level wavelet, and in EMD decomposition it is decomposed into two parts of the low-level mode and the high-level mode. The signal decomposition method proposed in this section hierarchical decomposes the sparse signal according to the component coefficients. If the mixed signal is sparse in the Fourier sparse basis, the part of low frequency and high frequency of the signal can be decomposed into one stage. Compared with traditional signal decomposition, this decomposition method is closer to the sparse characteristics of the information contained in the signal, and is suitable for compressed sensing applications. The main signal of the signal decomposed by this method is included in the signals of the first few levels, and the secondary signal is at a later level. Noise signals such as white noise signals are generally decomposed into the remaining residuals and eliminated.

**Figure 6:**
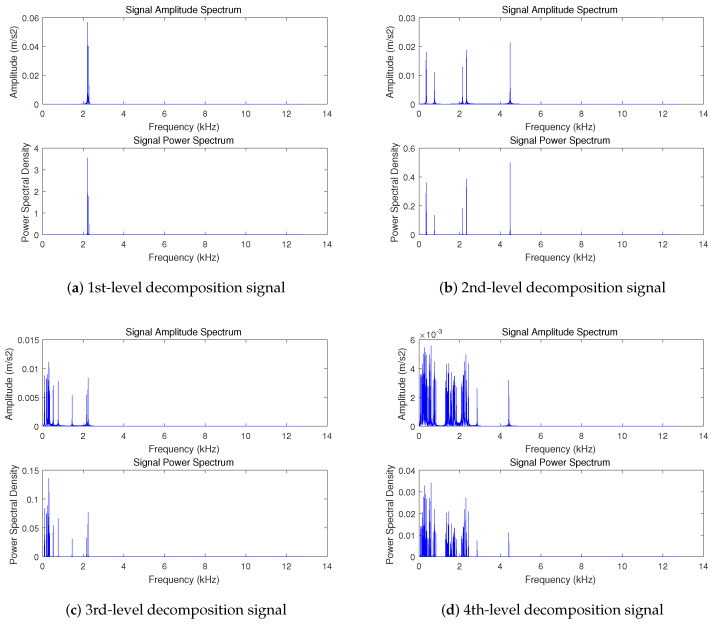
Amplitude spectrum and power spectrum of sub-signals.

This article uses vibration signals shown in Figure 4 as an example to show the process and results of hierarchical compression The sparse basis is used as the Fourier basis, and the adaptive grading threshold coefficient δ=0.5, the signal is divided into 4 levels. The result of decomposing the signal is as shown in Figure 6. The energy of the signal is gradually decreasing with each composition level, and the signal becomes less and less sparse. The sparsity of the sub-signal at each level starts from the first-level sub-signal as follows: {4,13,32,180}. According to Equation (3),under the condition of c=2.5, the value of $M_{i}$ is {35,94,197,741}.

**Figure 7:**
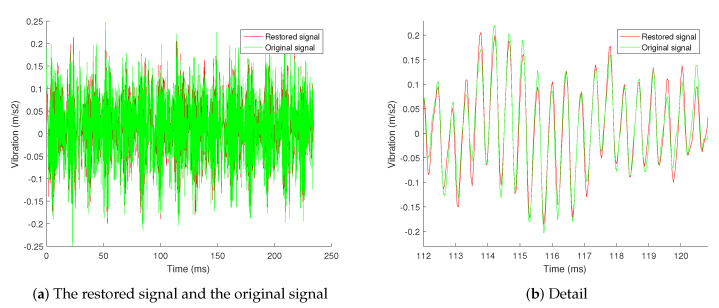
The comparison between the signal restored by the 4 sub-signals and the original signal.

**Figure 8:**
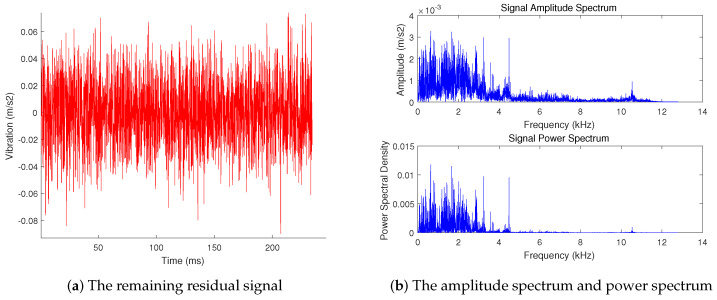
The remaining residual signal and the amplitude spectrum and power spectrum.

The comparison between the signal restored by the 4 sub-signals and the original signal is shown in Figure 7. The signal is not a complete restoration of the original signal, but an approximate restoration. The restored signal presents the main information of the original signal. The remaining residual signal and spectrogram are shown in Figure 8. The residual signal has no sparseness in the frequency domain, and its amplitude and energy are small. It only contains the secondary information and noise in the signal, which cannot be restored by the compressed sensing algorithm.

#### 3.2.3. Hierarchical Decomposition Method with the Different Sparse Bases (HDWDS)

**Figure 9:**
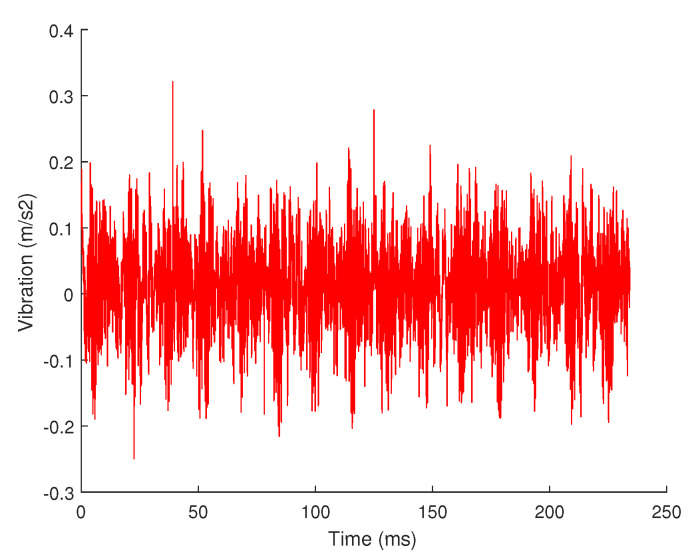
Vibration signal.

Many signals in nature may not only be sparse on a sparse basis. Take the vibration signal used in this article as an example, its waveform is shown in Figure 4 when it is working normally, but when it is near the end of its life, it may have sharp pulses, as shown in Figure 9.

**Figure 10:**
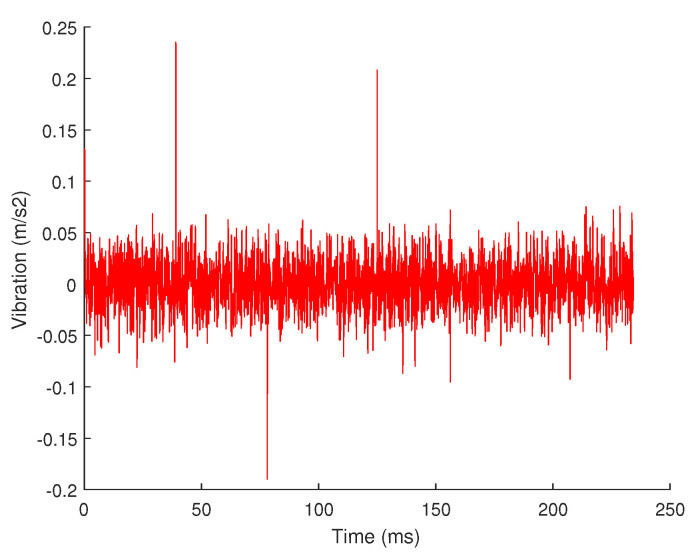
The residual signal obtained after the four-level decomposition.

These sharp pulse cannot be decomposed by the Fourier basis. The residual signal in the time domain obtained after the 4-level decomposition is shown in Figure 10. It can be seen that the residual signal after the 4-level FFT decomposition obviously contains these sharp pulse information. If the compressed sensing is performed according to the current result, the result will lose this part of information. Therefore, when designing the algorithm, we need to select the appropriate sparse basis according to the type of signal, including the same sparse basis or different sparse bases.

At the beginning of decomposition, the order of decomposition of sparse basis must be determined first. The order of the sparse basis is decomposed according to the main sparse domain of the current residual signal. The main sparse domain can be determined by determining the p-norm of the sparse vector of the signal under each sparse basis, and the sparse basis with the smallest p-norm is selected for signal decomposition of the main sparse domain. Then determine the main sparse domain of the residual residual signal after decomposition, and repeat the above steps. In practical applications, the sparse basis and its decomposition sequence can often be determined through prior knowledge. For example, the vibration signal superimposed on the instantaneous abnormal signal can be sparsely decomposed in the Fourier basis first, and then decomposed in the time domain. The main sparse domain can be determined by the smallest p-norm of the sparse vector of the current signal under each sparse basis.

**Figure 11:**
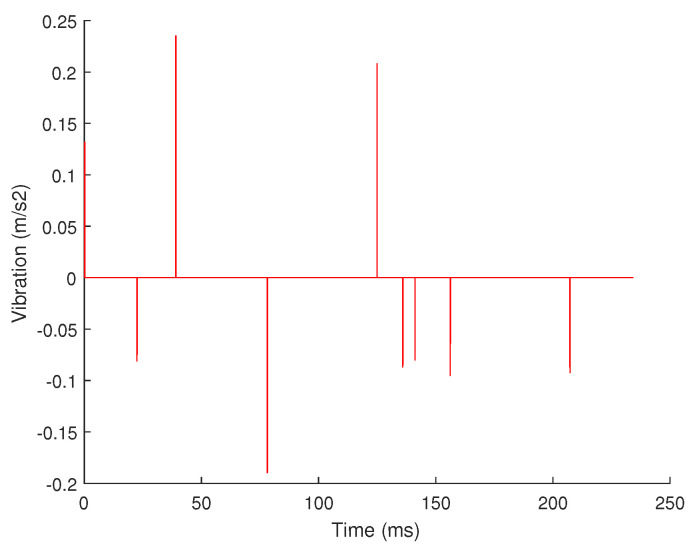
5th-level decomposition signal.

**Figure 12:**
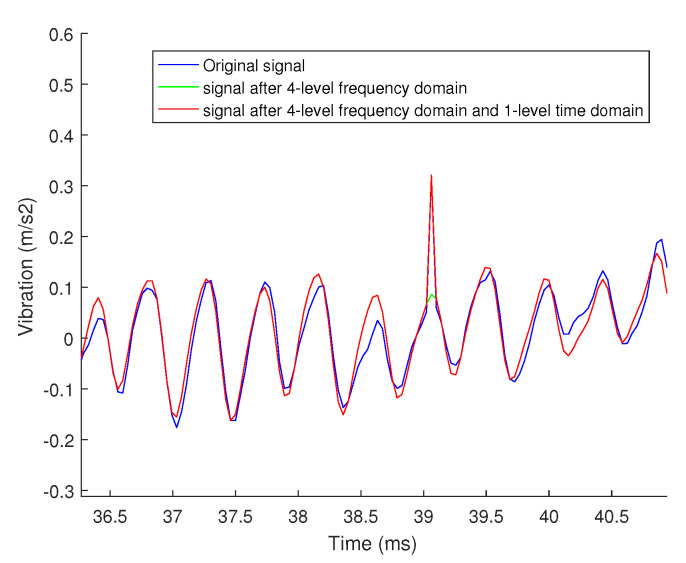
The results of decomposition and reconstruction with different sparse bases.

In this article, we added the 5-level time-domain decomposition on the basis of the above-mentioned 4-level decomposition, and selected the signals larger than a certain threshold in the time domain to form the 5-level sub-signals. The decomposition coefficient of the 5th grade signal is 0.5, and the decomposition result is shown in Figure 11. It can be seen from the figure that the 5th-level signal shows the sharp pulse signal in the time domain well. The final decomposition and reconstruction results of different sparse bases are shown in Figure 12. It is very obvious that the signal after the 4-level decomposition and reconstruction with Fourier basis loses the sharp pulse information in the original signal, while the signal after the 4-level decomposition and reconstruction with Fourier basis and time domain retains these informations well. Therefore, the hierarchical decomposition method with the different sparse bases greatly improves the accuracy of reconstruction.

### 3.3. Hierarchical Compressed Sensing Method with a Mask (HCSWM)

#### 3.3.1. Create an Observation Matrix

It can be seen from the signal decomposition method that the sparsity of the signals at all levels is different. The sub-signals with a lower number of layers are more sparse, and the M value required for the observation matrix is smaller. If the observation matrix is defined separately for each layer of sub-signal, it is easy to cause a waste of hardware resources. In order not to save the redundant observation matrix, we first save the same M value of the total observation matrix [$M_{max},N$] at the network node and terminal software according to the prior knowledge, and satisfy

(19) $$\begin{array}{c} M_{max}>\max\left(M_{1},M_{2}\cdots M_{n}\right) \end{array}$$

The observation matrix is obtained by multiplying a sufficiently large Bernoulli random matrix with the prior sparse basis of the signal. The number of columns of the matrix is the same as that of the total observation matrix, and the number of rows is based on the $M_{i}$ required by each level of sub-signal, from the first row to the $M_{i}$-th row of the total observation matrix. In this way, the terminal can determine its observation matrix according to the dimensions of the received sub-signals, and then perform subsequent data reconstruction.

From the perspective of space occupation, the hierarchical compression method can choose to share the observation matrix, so the space occupied by the hierarchical compression method is much smaller than that of the traditional compressed sensing method. The traditional compressed sensing method needs to occupy [N,M] space to store the observation matrix, and the proposed method only needs to occupy [$N,max\left(M_{i}\right)$] space. At the same time, as the matrix decreases, the space occupied by temporary variables in the calculation process will also decrease.The ratio $R_{s}$ of the space occupied by our proposed method and the traditional method is

(20) $$\begin{array}{c} R_{s}=max\left(M_{i}\right)/M \end{array}$$

#### 3.3.2. Design the Mask of the Observation Matrix

Due to the sparseness of the signal, for a certain sub-signal, because most elements of the sparse signal are 0, there are a few column vectors in the observation matrix that actually participate in the calculation during the compression coding process. In addition, for natural physical quantity signals, after sparse transformation in some common domains (such as frequency domain, wavelet domain, etc.), the index positions of non-zero values in the sparse signals are often close or adjacent. As shown in Figure 6, it can be seen that the vibration signal is approximately sparse in the frequency domain, and for each sub-signal, most of the sparse signals are 0, and non-zero values are mostly concentrated in a few fixed areas. (For example, vibration signals are mostly concentrated in one or more limited frequency bands). To this end, we design a mask Λ of the observation matrix to mark the approximate area or range of the atoms in the observation matrix that participate in the calculation during encoding. The detailed process of mask participating in the calculation is shown in Algorithm 2. The mask is transmitted to the terminal together, which can reduce the number of atoms participating in projection in the observation matrix during reconstruction, reduce the amount of calculation during reconstruction.

**Figure 13:**
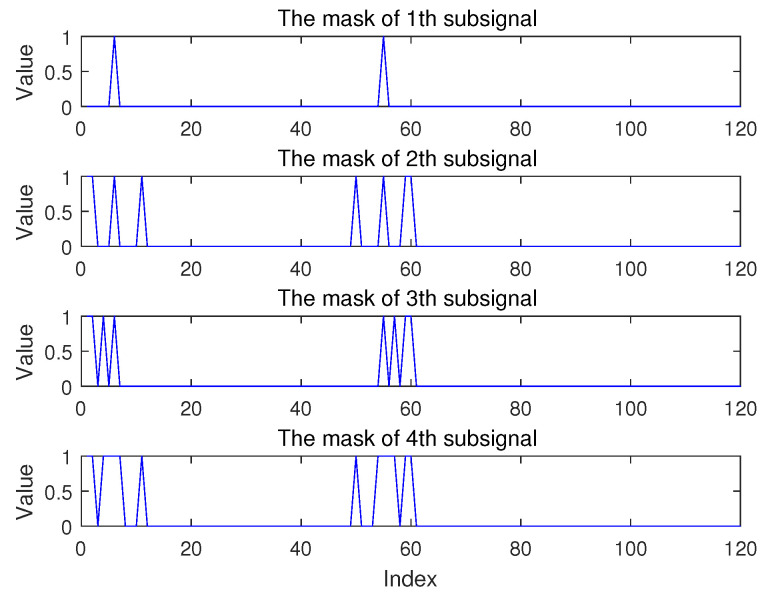
The mask of 4 sub-signal.

To reduce the data length of the mask vector, the atoms in each mask do not indicate whether the atoms of a single observation matrix participate in encoding, but correspond to a continuous set of atoms of the observation matrix. 0 indicates that there is no atom involved in the calculation in the corresponding observation matrix, 1 indicates that there is an atom involved in the encoding calculation in the corresponding observation matrix. Each element represents 50 data and can be represented by 1 bit. The sum of all elements is 6000/50/8=15 bytes, which is only equivalent to 7.5 data. Therefore, the length of the mask has little effect on the compression ratio. Take the sub-signal shown in Figure 6 as an example, the mask is as shown in Figure 13.

#### 3.3.3. Sub-Signal Hierarchical Compression

The compressed signal is obtained by Equation (21)

(21) $$\begin{array}{c} C_{i}=\Phi D_{i} \end{array}$$

#### 3.3.4. OMP Algorithm with a Mask

As a classic algorithm for signal reconstruction in compressed sensing, OMP algorithm is widely used in various application scenarios [28,29]. In contrast to the traditional OMP algorithm, the OMP algorithm designed in this paper with a mask, an observation matrix and signal layering method to improve the efficiency of the algorithm. The specific steps are shown in Algorithm 2.

Because the signals in nature are generally concentrated in certain continuous frequency domains or other domains, most of the remaining signals are 0 or close to 0. The function of the mask we designed is that only non-zero signals participate in the calculation, which greatly reduces the amount of calculation. Taking the mask in Figure x as an example, only the elements corresponding to 2 atoms in the 1st subsignal participate in the calculation, and 8, 8, 14, 9 in 2nd, 3rd, 4th, 5th subsignal. The atomic sum of the 5 subsignal involved in the calculation is 41. The mask can reduce the amount of calculation to 34% of the original (Only refers to the calculation amount of line11 in Algorithm 2). The advantage of using hierarchical reconstruction of the signal is that after the transmission of a single sub-signal, the original signal can be reconstructed and output. With the more sub-signals reconstructed, the more detailed information of the original signal and the smaller the distortion. This hierarchical reconstruction method is different from the traditional signal reconstruction, and the reconstructed signal can be output without receiving all the compressed information.

**Algorithm 2** OMP algorithm based on a mask and observation matrix
**input**: Observation matrix Φ; compressed signal $C_{i}$; mask Λ;
**output**: recovered sparse sub-signals $Y_{i}$
1:eachmask = 50, level = 4;2:fori=1:1: level3:   $T=\Phi_{i}\Psi^{T};$4:   $product=Ø;Aug_{t}=Ø;posa=Ø;tmpy=Ø$5:   $r_{n}=\hat{Y_{i}};Nd=a*M_{i};$6:   fortimes=1:1:Nd;7:      forcol=1:N;8:         $if(mask_{i}\left(ceil\left(col/eachmask\right)==0\right)$9:            product(col)=0;10:         else11:            $product\left(col\right)=abs(T{\left(:,col\right)}^{T}*r_{n});$12:      [val,pos]=max(product);13:      $Aug_{t}=\left[Aug_{t},T\left(:,pos\right)\right];$14:      $Aug_{y}=\left(Aug_{t}^{T}*Aug_{t}\right)\setminus \left(Aug_{t}^{T}*\hat{Y_{i}}\right);$15:      posa(times)=pos;16:   $Y_{i}\left(posa\right)=Aug_{y};$17:   $Y=Y+Y_{i};$


**Figure 14:**
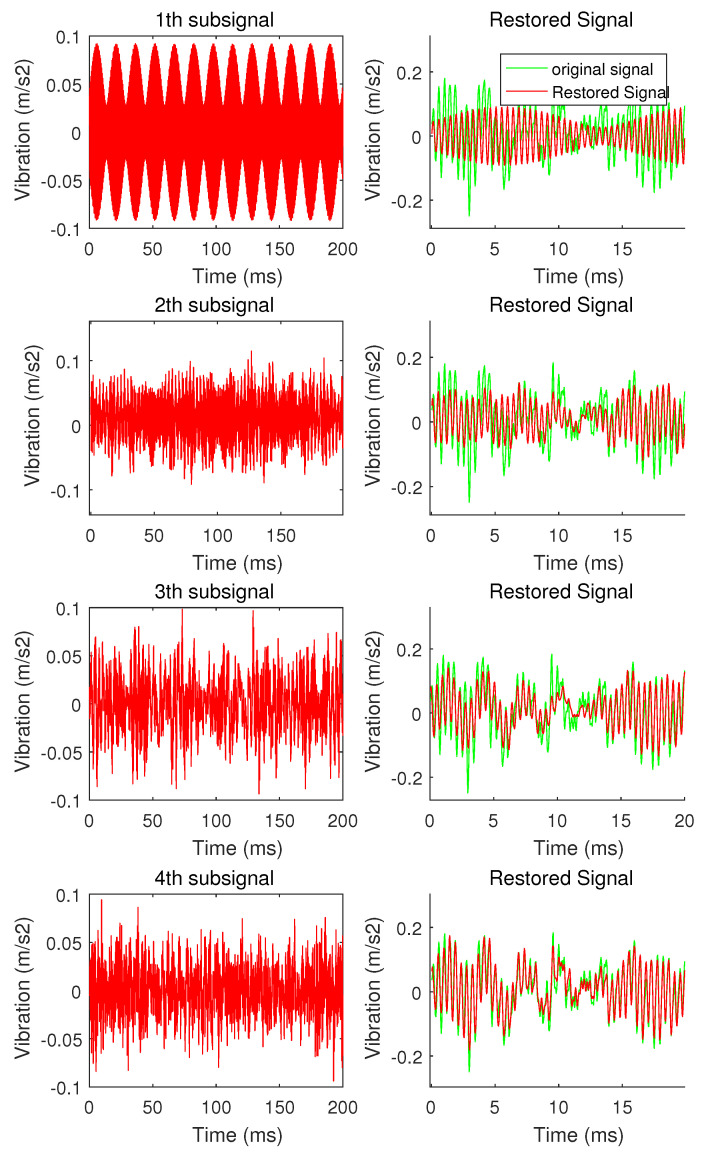
Amplitude spectrum and power spectrum.

In this way, the reconstructed signal output process transforms the process of the traditional compressed sensing from “nothing” to “being” into a process from “fuzzy” to “clear”. The restoration process is shown in Figure 14. The compression ratio of the first three levels of signal is 5.01%, which restores the trend change of the original signal; The compression ratio of the first four levels of signal is 16.45%, which can completely restore the sparse signal *O*.

To analyze the advantages of the proposed method in terms of the amount of calculation, we analyze the amount of calculation separately from the compression side and the reconstruction side. The traditional compressed sensing algorithm mainly performs matrix multiplication operations of [1,N] and [N,M] on the compression side, and the method we proposed performs matrix multiplication operations of [1,N] and [$N,M_{1}$], [$N,M_{2}$]...[$N,M_{n}$], the calculation amount $C_{t1},C_{o1}$ on the compression side is shown in the following formula:

(22) $$\begin{array}{cc} C_{t1} & =N^{2}*M\\ C_{o1} & =N^{2}*M_{1}+N^{2}*M_{2}+\cdots +N^{2}*M_{n}\\ M & =M_{1}+M_{2}+\cdots +M_{n} \end{array}$$

Therefore, the traditional compressed sensing on the compression side is basically the same as the method proposed in this article:

(23) $$\begin{array}{c} C_{t1}=C_{o1} \end{array}$$

The calculation amount of the traditional algorithm and the algorithm we proposed on the reconstruction side is mainly related to the OMP algorithm. The calculation amount of the OMP algorithm is mainly related to the number of iterations (Nd) and the calculation amount (Cd) during each iteration. Nd and Cd are positively correlated with *M* [30].

(24) $$\begin{array}{cc} Nd & =a\times M\\ Cd & =b\times M \end{array}$$

where a and b are coefficients. Then the calculation amount $C_{t2}$ of the traditional compressed sensing algorithm on the reconstruction side and the calculation amount $C_{o2}$ of The traditional compressed sensing algorithm can be shown as:

(25) $$\begin{array}{cc} C_{t2} & =Nd\times Cd\\ \; & =a\times M\times b\times M\\ C_{o2} & =Nd\times Cd\\ \; & =a\times M_{1}\times b\times M_{1}\\ \; & +a\times M_{2}\times b\times M_{2}\\ \; & +\cdots \\ \; & +a\times M_{n}\times b\times M_{n} \end{array}$$

It is very obvious that:

(26) $$\begin{array}{c} C_{t2}>>C_{o2} \end{array}$$

The calculation amount of our proposed method is far less than the traditional compressed sensing algorithms.

### 3.4. Packet Structure and Management

**Figure 15:**
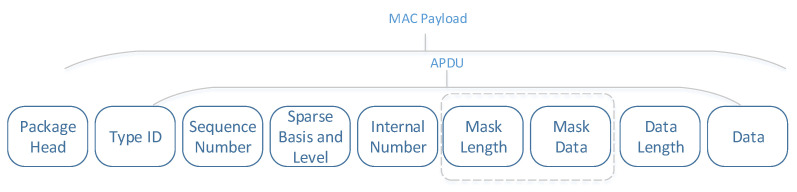
Packet structure.

Due to the packet loss phenomenon in wireless sensor networks, we have designed a robust wireless network packet structure, as shown in Figure 15 and the lost data will not affect the subsequent data reconstruction as much as possible. Each data packet is relatively independent, and contains information such as signal level, sparse basis type, and packet number.

**Figure 16:**
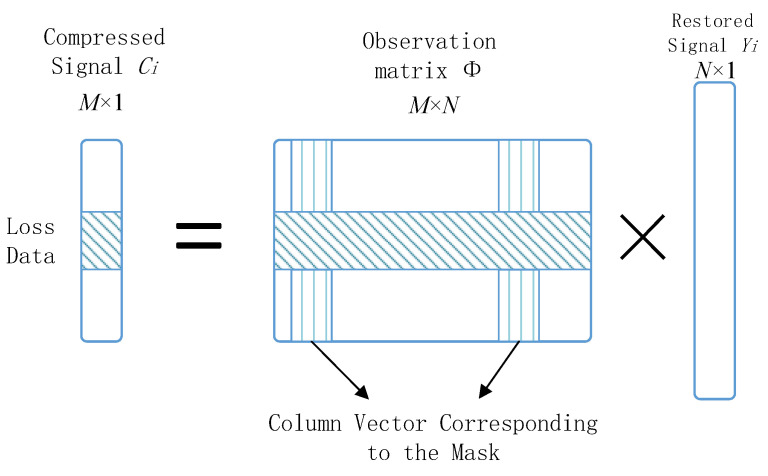
Correspondence between each encoded data packet and the row vector of the observation matrix.

Each encoded data packet corresponds to a series of continuous row vectors of the observation matrix, as shown in Figure 16. The shaded area in the compressed data is the lost data packet. The shaded area in the observation matrix is the corresponding row vector. The row vector of the observation matrix involved in the reconstruction calculation is determined by the row vector corresponding to the sequence number and length of the received data packet. Therefore, a small amount of packet loss will not affect the operation of the reconstruction algorithm. At the same time, we can use the number of sub-signal levels for priority management. Data with lower levels has higher priority in routing and buffering, which increases the robustness of the network and reduces the possibility of loss of important information. Therefore, the algorithm proposed in this paper is also more robust compared with other sparse coding techniques.

## 4. Comparative Experiment

### 4.1. Execution Time Comparison Experiment

To verify that the algorithm proposed in this article has advantages in terms of calculation amount, we designed an execution time comparison experiment with the traditional compressed sensing algorithm, the hierarchical compressed sensing algorithm and the hierarchical compressed sensing algorithm with a mask. The three experiments use the same original data, as shown in Figure 4. In addition, the sparsity of traditional compressed sensing algorithms is equal with the sum of sparsity of multi-level signals in the hierarchical compressed sensing method, and is equal with the hierarchical compressed sensing algorithm with a mask, to ensure the same compression ratio. The hierarchical compressed sensing algorithm and the hierarchical compressed sensing algorithm with a mask both use a 4-level decomposition under the same condition of c=2.5,δ=0.5. The results of the execution time of different methods are shown in Table 1.

In this experiment, under the same compression ratio, the hierarchical compressed sensing method saves about 24% of the time compared with the traditional compressed sensing and the hierarchical compressed sensing method with a mask saves about 64% of the time than the traditional compressed sensing method.

### 4.2. Comparison Experiment of Compression Ratio and Compression Effect

Aiming at the compressed sensing algorithm designed in this paper, we designed a comparison experiment with the traditional compressed sensing method and the hierarchical compressed sensing method with mask under the different compression ratio. Traditional compressed sensing and hierarchical compressed sensing method with a mask are difficult to compare under exactly the same conditions. Therefore, the hierarchical compressed sensing algorithm with a mask uses a 5-level decomposition under the condition of c=2.5 and different δ to achieve different compression ratios, and the traditional compressed sensing method used different $K_{\mathrm{sp}}$ to achieve different compression ratios. The calculation of reconstruction error adopts the following formula:

(27) $$\begin{array}{c} Error=norm(Y-O)/norm(O) \end{array}$$

**Figure 17:**
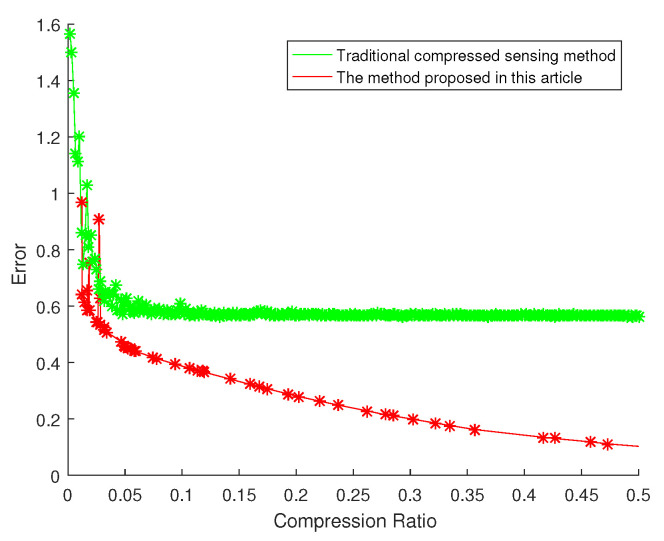
Experimental results of different methods.

The experimental results of different methods are shown in the Figure 17. It can be seen that the algorithm proposed in this article has lower error performance under the same compression rate. Under the condition of 5% compression rate, the algorithm proposed in this paper reduces the error by 26% compared with the traditional compressed sensing algorithm. Under the condition of 10% compression rate, the algorithm proposed in this paper reduces the error of the traditional compressed sensing algorithm by 35%. Under the condition of 15% compression rate, the algorithm proposed in this paper reduces the error of 43% compared with the traditional compressed sensing algorithm.

## 5. Discussion

### 5.1. Disscussion on Execution Time

According to the analysis of Section 3.3.4 in this article, the hierarchical compressed sensing method and mask method proposed in this article can greatly reduce the amount of calculation. Thus, compared with the traditional compressed sensing methods, the method proposed in this paper has certain advantages in execution time as shown in Table 1.

### 5.2. Disscussion on Compression Ratio and Reconstruction Error

Compressed sensing algorithm can restore the original signal very well when satisfying the condition of Formula (10). However, the signal in nature is generally not strictly sparse, and the non-sparse part of the original signal will affect the result, causing its error to be relatively large. Thus, as the compression rate increases but the error remains stable. The algorithm proposed in this paper uses sparse decomposition to remove the non-sparse parts of the original signal in advance, so that our method has a better reconstruction effect.

### 5.3. Disscussion on Resource Occupation

The method proposed in this paper shares the observation matrix, so the resource occupied is much smaller than that of the traditional compressed sensing method. At the same time, as the matrix decreases, the space occupied by temporary variables in the calculation process will also decrease.

### 5.4. Discussion on Other Advantages of Compressed Sensing Methods

Traditional compressed sensing has the advantages of automatic encryption, high compression rate, small reconstruction error, and insensitive to packet loss. The method proposed in this article retains these advantages. This paper designs the packet structure of wireless transmission for large data, and implements priority management based on the number of hierarchical layers, which further improves the robustness of signal reconstruction.

## 6. Conclusions

To reduce the amount of data transmitted in the wireless sensor network, we proposed a wireless transmission method for large data based on hierarchical compressed sensing and sparse decomposition. This method includes a hierarchical signal decomposition method based on the same sparse basis and different sparse basis hierarchical compressed sensing method with a mask. Compared with the traditional compressed sensing algorithm, this method has lower signal reconstruction error, lower resource consumption, lower calculation amount, can realize data reconstruction while displaying, and retains the advantages of automatic encryption, high compression rate, small reconstruction error, and insensitive to packet loss. Thus, this method is more suitable for wireless sensor networks than traditional compressed sensing methods. We designed the packet structure and implemented priority management, which further improved the robustness of signal reconstruction. Finally, it is verified through experiments that the algorithm proposed in this paper has a better data reconstruction effect at a relatively low compression ratio and a lower execution time compared with the traditional compressed sensing algorithm.

## Figures and Tables

**Table 1:** Execution time of different methods.

Method	Traditional Compressed Sensing	Hierarchical Compressed Sensing Algorithm without Mask	Hierarchical Compressed Sensing Algorithm with Mask
execution time(s)	7.3354	5.5780	2.6598

## Abbreviations

The following abbreviations are used in this manuscript:

OMP	Orthogonal matching pursuit
HCSWM	Hierarchical compressed sensing method with a mask
HDWDS	Hierarchical decomposition method with the different sparse bases
HDWSS	Hierarchical decomposition method with the same sparse bases
MDPI	Multidisciplinary Digital Publishing Institute
DOAJ	Directory of open access journals
TLA	Three letter acronym
LD	Linear dichroism
